# An Overview of the Molecular Mechanisms Associated with Myocardial Ischemic Injury: State of the Art and Translational Perspectives

**DOI:** 10.3390/cells11071165

**Published:** 2022-03-30

**Authors:** Leonardo Schirone, Maurizio Forte, Luca D’Ambrosio, Valentina Valenti, Daniele Vecchio, Sonia Schiavon, Giulia Spinosa, Gianmarco Sarto, Vincenzo Petrozza, Giacomo Frati, Sebastiano Sciarretta

**Affiliations:** 1 Department of Medical and Surgical Sciences and Biotechnologies, Sapienza University of Rome, 04100 Latina, Italy; leonardo.schirone@uniroma1.it (L.S.); l.dambrosio@uniroma1.it (L.D.); valevale2012@hotmail.com (V.V.); daniele.vecchio@uniroma1.it (D.V.); sonia.schiavon@uniroma1.it (S.S.); giu.spi15@gmail.com (G.S.); sarto.gianmarco@gmail.com (G.S.); vincenzo.petrozza@uniroma1.it (V.P.); fraticello@inwind.it (G.F.); 2 IRCCS Neuromed, 86077 Pozzilli, Italy; maurizio.forte@neuromed.it; 3 Istituto Pasteur Italia—Fondazione Cenci Bolognetti and Department of Medical and Surgical Sciences and Biotechnologies, Sapienza University of Rome, 04100 Latina, Italy

**Keywords:** ischemic injury, myocardial ischemia, infarction, ischemia/reperfusion (I/R), molecular, mechanisms, pathway, translational, state of the art

## Abstract

Cardiovascular disease is the leading cause of death in western countries. Among cardiovascular diseases, myocardial infarction represents a life-threatening condition predisposing to the development of heart failure. In recent decades, much effort has been invested in studying the molecular mechanisms underlying the development and progression of ischemia/reperfusion (I/R) injury and post-ischemic cardiac remodeling. These mechanisms include metabolic alterations, ROS overproduction, inflammation, autophagy deregulation and mitochondrial dysfunction. This review article discusses the most recent evidence regarding the molecular basis of myocardial ischemic injury and the new potential therapeutic interventions for boosting cardioprotection and attenuating cardiac remodeling.

## 1. Introduction

According to the World Health Organization, cardiovascular diseases (CVDs) are the leading cause of death in western countries. They account for 30% of deaths worldwide, killing over 17 million people every year [1]. The increase in life expectancy and the spread of cardiovascular risk factors (e.g., obesity, diabetes, metabolic syndrome) in young people are causally linked to the increasing incidence of CVDs [2]. For this reason, in 2015, the United Nations General Assembly published the Sustainable Development Goals (SDGs) to reduce the incidence of death due to non-communicable diseases, including CVDs, by one-third by 2030 [3]. To achieve this goal, the implementation of political strategies, economic interventions and research funding is warranted [1].

In past decades, much effort has been invested in the characterization of the molecular mechanisms underlying the development of ischemic damage. This review summarizes the current knowledge of the molecular mechanisms underlying myocardial ischemia and discusses new potential therapeutic interventions for reducing acute myocardial ischemic injury and post-ischemic cardiac remodeling.

## 2. Pathophysiology of Myocardial Infarction

The heart requires an extensive energy supply to maintain its function, which is provided by adenosine triphosphate (ATP), mainly produced through oxidative phosphorylation. Cardiomyocytes are strictly dependent on oxygen to meet their metabolic demands. An interruption of coronary blood flow rapidly stresses cardiomyocytes and ultimately leads to apoptosis and necrosis [4]. The elucidation of mechanisms involved in heart infarction is crucial for finding new therapeutic targets to attenuate ischemic injury, particularly considering that the myocardium is a tissue with extremely low regenerative potential. These include interventions to reduce cell death and mitigate inflammatory and metabolic derangements. Novel biotechnological strategies based on pluripotent stem cells, hydrogels and nanotechnology may also be potentially efficacious to regenerate myocardial scarring (see Section 8).

### 2.1. Causes of Ischemic Heart Disease

Conditions leading to myocardial ischemia in patients may occur both acutely and chronically. These conditions are characterized by a lack of balance between demand and supply of oxygen and nutrients to myocardial tissue. Acute myocardial ischemia usually involves the rapid reduction or arrest of coronary blood flow to the myocardium, which impairs cardiomyocyte function and survival, eventually leading to myocardial infarction (MI) if blood flow is not rapidly restored. It is commonly caused by an athero-thrombotic event that partially or entirely occludes the coronary artery [5]. On the other hand, chronic myocardial ischemia is a prolonged reduction of oxygen supply due to significant and chronic coronary artery blood flow reduction, which causes low-magnitude cumulative injury at resting conditions due to compensatory vasodilation of coronary arteries. Conversely, myocardial ischemia usually occurs during effort and in other conditions increasing oxygen demand because of the failure of compensatory coronary vasodilation to meet metabolic cardiomyocyte needs [5]. Chronic myocardial ischemia conditions are usually caused by stable coronary atherosclerotic plaques that reduce coronary blood flow significantly.

In general, ischemic heart disease can be divided into vascular and non-vascular causes [5].

Vascular causes include:

- Coronary artery atherosclerosis

Increased low-density lipoproteins (LDL), reactive oxygen species (ROS) and inflammation promote vascular inflammation sustained by foamy macrophages that stratify below the permeabilized endothelium and, together with myofibroblasts and other cell types (e.g., lymphocytes, mast cells, neutrophils, endothelial cells), form an atherosclerotic plaque [6]. Besides, diabetic-associated hyperglycemia enhances the formation of advanced glycosylation products (AGEs), which cause an increase in inflammatory endothelial patterns. Consistently, both acute and chronic infections increase vascular inflammation, stimulating atherosclerotic remodeling and monocyte infiltration [5].

- Coronary artery spasm

This term indicates intense vasoconstriction of the epicardial coronaries that causes a vascular sub-occlusion or a complete occlusion. Although this pathological event may cause acute ischemia, repetitive episodes of vasospasm-induced ischemia may eventually contribute to the development of chronic ischemic myocardial abnormalities. The mechanisms underlying coronary spasm include endothelial dysfunction, smooth muscle cells hyperactivity and other factors [7]. Endothelial damage causes a reduction in the levels of nitric oxide synthase (NOS) that causes an increase of vascular tone, which also determines changes in endothelial smooth muscle cells. Among these, the increased activity of Rho-kinase and protein kinase C (PKC) promote hyper-contraction by increasing Ca^2+^ sensitivity and by inhibiting myosin light chain phosphatase (MLCP) through PKC-mediated phosphatase inhibitor (CPI-17) and myosin phosphatase target subunit 1 (MYPT-1) phosphorylation [8]. Hyperactivity of these mechanisms underpins coronary spasms. Other potential triggers for the development of coronary spasms are represented by drug stimulation (e.g., cocaine), eccentric stenosis (which causes an increase in vascular tone), fibromuscular hyperplasia, adventitial abnormalities such as infiltration of inflammatory cells, and impaired calcium release. The latter impairs the contractile function of smooth muscle cells [9].

- Microvascular dysfunction

One of the most common mechanisms for developing microvascular dysfunction is altered endothelial vasodilation caused by a reduction of nitric oxide bioavailability due to a reduced response to acetylcholine. Different studies have shown an increase in vasoconstriction activity [10]. In addition, ventricular hypertrophy and dilation associated with genetic cardiomyopathies, arterial hypertension or aortic stenosis reduce the density of coronary capillary vessels and/or increase the intensity of extravascular compressive forces. These phenomena, together with inflammation, promote vascular fibrosis and dysfunction. The main risk factors related to the onset of this condition include cigarette smoke, which is responsible for an increase in oxidative stress [11,12,13,14], hyperlipidemia, and diabetes, which may promote vasoconstriction. Overall, these conditions may cause microvascular angina, with the formation of areas of micro-infarctions due to a reduction of coronary flow reserve [15].

- Vascular remodeling

Oxidative stress associated with inflammatory mechanisms and endothelial damage determines vascular remodeling. This can be either an adaptive response, with a widening of the external elastic membrane to stabilize the vessel lumen in the atheromatous area, or a maladaptive response. The latter involves a failed compensation of the obstruction, which reduces the lumen below 70% with the consequent loss of perfusion capacity [5,16].

Non-vascular causes of myocardial ischemia include cardiac hypertrophy, cardiac remodeling and dysfunction, hematological diseases, especially anemia, traumas and pulmonary diseases.

### 2.2. Ischemia/Reperfusion Injury

**Figure 1:**
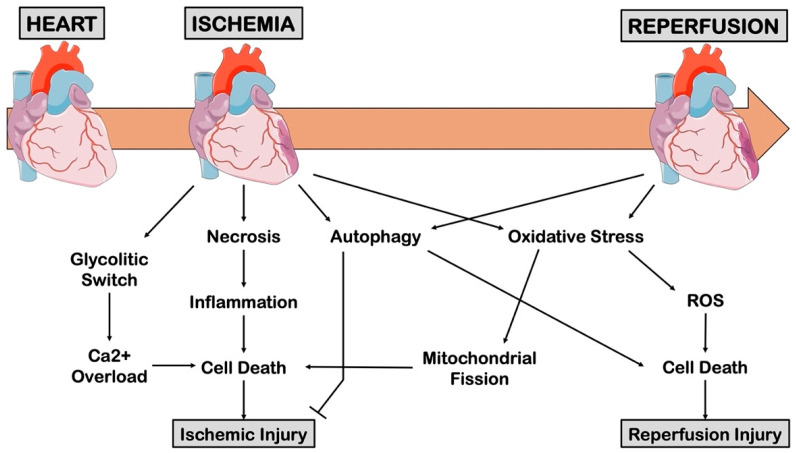
Schematic representation of the molecular mechanisms contributing to ischemia and reperfusion injury. The figure was made in part using tools provided by Servier Medical Arts.

Restoration of coronary blood flow is essential to save the life of patients with acute MI and reduce myocardial loss. However, myocardial reperfusion also contributes to the extension of MI through excessive production of ROS, intracellular calcium overload and mitochondrial damage, namely the reperfusion injury. Therefore, when pathophysiological mechanisms underlying acute myocardial ischemia are analyzed, it is mandatory to distinguish the ischemic and reperfusion phases since the molecular events leading to myocardial injury in these phases are significantly different (Figure 1).

The concept of biphasic ischemia/reperfusion (I/R) injury is now well established. Although vasodilation of coronary arteries up to three to five times above basal levels can compensate for blood flow reduction due to stable atherosclerotic plaques, acute atherothrombosis leads to a sudden and dramatic interruption of oxygen and nutrient supply to the myocardium, which determines the interruption of mitochondrial oxidative phosphorylation (as detailed below) within fifteen to twenty seconds [17]. This event causes a drastic reduction of ATP availability, which reduces myocardial contractile function just a few minutes after the onset of ischemia, causing the so-called ‘heart rigor contracture’ [18]. The reduction of ATP production becomes almost complete (90–95%) after 40–60 min of ischemia [19]. This is considered the “point of no-return” for cardiomyocyte death and the occurrence of irreversible structural changes in the myocardium [20]. Beyond cardiomyocytes, reperfusion is also associated with other detrimental mechanisms, such as activation of the complement system due to accumulated Immunoglobulin M (IgM) in ischemic tissue. It was previously observed that IgM blockage exerts protective effects and improves cardiac function after acute myocardial ischemia in a murine model of ischemic damage [21]. Besides, increased ROS production (by cardiomyocyte, stromal and inflammatory cells) and inflammation are major players of reperfusion-mediated injury and are discussed in detail in later sections [22].

Reperfusion reintroduces the substrates for ATP generation and oxygen, allowing pH normalization and the wash-out of catabolic wastes. These factors are of paramount importance for the survival of myocardial tissue, but in cardiomyocytes with a significant ischemic insult the same factors may paradoxically exacerbate cell injury, determining the transition from reversible to irreversible damage. Reperfusion induces a rapid normalization of the extracellular pH that determines the formation of a strong H^+^ gradient across the plasma membrane. The main result is a further massive flow of Na^+^ inside the cell to enable the expulsion of excess H^+^ ions through the Na^+^/H^+^ exchanger. The increase of intracellular Na^+^ forces the sodium-calcium exchanger (NCX) to operate unconventionally, secreting Na^+^ outside the cell to balance its accumulation and importing Ca^2+^ [23]. It takes 30–60 min to reestablish physiological calcium levels, and the transient accumulation of this ion in the cytoplasm is sufficient to activate calcium-dependent lipases and proteases and trigger cell hyper-contraction and mitochondrial permeability transition pore (mPTP) opening [24]. Even though the exact composition of the mPTP core remains to be fully clarified, an increasing consensus is gathering around a structural role for adenosine nucleotide transporter (ANT) [25] and F_0_F_1_-ATP synthase [26]. Voltage-dependent anion-selective channel (VDAC), hexokinase, creatine kinase, B-cell lymphoma 2 (Bcl-2), Bcl-2 homologous antagonist killer (Bak), Bcl-2-associated X (Bax), Bcl-xL, cyclophilin D, benzodiazepine receptor, glycogen synthase kinase 3 beta (GSK3-β), PKCε, protein kinase G (PKG), p53 and the complement component 1 Q subcomponent-binding protein (C1QBP) also play a regulatory role in mPTP opening [27]. mPTP transient opening in a low-conductance (<300 KDa) mode is physiological and permits calcium efflux, redox signaling and cardiomyocyte development. However, a prolonged or higher conductance opening leads to harmful effects, including mitochondrial depolarization, swelling and massive release of cytochrome C and Ca^2+^, which trigger the intrinsic apoptotic pathway and necrosis, respectively [28]. We recommend consulting the several review articles on the mechanisms of I/R [19,29] and the role of mitochondria in I/R [30,31,32] that were previously published. However, new studies and discoveries on I/R-induced cell death mechanisms persuaded us to implement these in a comprehensive synthetic dissertation on the currently known mechanisms underlying I/R injury in the heart.

### 2.3. Chonic Post-Infarction Remodeling

I/R injury (hereafter referred to as acute myocardial ischemia in some cases in the text) and non-reperfused myocardial ischemia (hereafter referred to as chronic MI when animal models of chronic coronary ligation are discussed) lead to the development of MI, with a considerable myocardial loss and the development of cardiac dysfunction. Cardiac dysfunction triggers a complex process of myocardial derangements, including myocyte loss, fibrosis, wall thinning, ventricular dilation, metabolic alterations, mitochondrial dysfunction, dysregulated autophagy and mitochondrial dynamics. This maladaptive myocardial response contributes to the so-called post-infarction myocardial remodeling, with a progressive impairment of contractile function and eventually heart failure. For a deeper and more detailed description of the specific molecular mechanisms underlying cardiac remodeling and heart failure, we suggest consulting other review articles focused on this topic [33].

### 2.4. Cell Death in Myocardial Ischemic Injury

Cardiac cell death may occur through different mechanisms in response to myocardial ischemic injury [31]. Apoptosis may be triggered by the mitochondrial release of cytochrome C, which in turn activates the caspase-mediated intrinsic apoptotic pathway. Activating death receptors on the cell surface (e.g., tumor necrosis factor receptor 1 (TNFR1)) may also promote apoptosis in this context. Depending on the composition of the protein complex binding to the cytosolic region of death receptors, necroptosis may also be triggered in place of apoptosis if phosphorylated receptor-interacting protein kinase 3 (P-RIPK3) is recruited by receptor-interacting protein kinase 1 (RIPK1) in the so-called ‘necrosome complex’. P-RIPK3, in turn, activates the mixed lineage kinase-like domain (MLKL), a pseudokinase that mediates plasma membrane permeabilization [34]. This regulated form of necrosis is preferentially triggered in heavily inflamed tissues in the absence of active Caspase 8, which is known to cleave the key necroptotic protein RIPK 1 [35]. Systemic RIPK3-KO [36] and allosteric RIPK1 inhibition by necrostatin-1s [37] were both demonstrated to reduce infarct size in murine models of I/R injury. In contrast, the double systemic KO of TNFR1 and TNFR2 exacerbates infarct size, probably by the impairment of the immune response to myocardial non-reperfused injury [38].

Besides apoptosis and necrosis, several lesser-known mechanisms of regulated cell death are involved in ischemic damage. These include forms of cell death that are regulated by autophagy (autosis, discussed in Section 6), inflammation (pyroptosis) and iron (ferroptosis). Pyroptosis is a form of cell death triggered by intense activation of the inflammasome by dysfunctional mitochondria and activated Toll-like receptors and TNFR1 signaling. This leads to the activation of Caspase-1, 4 and 5, which cleave gasdermin D (GSDMD) to its active form. The latter contributes to the formation of pores in the plasma membrane by homopolymerization [39]. Global pro-caspase-1 gene deletion reduced infarct size in a murine model of I/R [40] and ameliorated the phenotype of ischemic cardiac remodeling and heart failure in mice [41]. Ferroptosis is a form of cell death triggered by membrane lipid peroxidation favored by inhibition of glutathione peroxidase 4 (GPX4) and Fe^3+^-mediated activation of lipoxygenase [42]. Oxidized lipids were also found to be increased in models of I/R, although the exact mechanism by which they cause cell death still needs to be clarified [43]. Moreover, inhibition of ferroptosis by Ferrostatin1 administration [44] or systemic overexpression of a mitochondrial-targeted mutant GPX4 [45] reduced infarct size in models of murine I/R and ex vivo perfused hearts, respectively. However, these studies did not provide mechanistic evidence about a direct involvement of ferroptosis, nor quantified the level of ferroptosis markers. Only a few reports investigated this aspect. For example, in mice subjected to I/R, Li et al. found increased levels of ferroptotic oxidized phosphatidylethanolamine (oxPE), key mediators of ferroptosis. They further demonstrated that Ferrostatin-1 decreases levels of hydroperoxy-arachidonoyl-phosphatidylethanolamine along with reducing infarct size and improving cardiac function [46]. Rat hearts undergoing I/R ex-vivo showed increased levels of ferroptotic oxPE in mitochondria and reduced mitochondrial bioenergetics, suggesting a central role of these organelles in ferroptotic cell death [47]. Of interest, Sparvero et al. provided direct evidence about the spatial distribution of ferroptotic oxPE in cardiac myoblast H9c2 undergoing ferroptotic stimuli, showing a marked accumulation in the endoplasmic reticulum, mitochondria and mitochondria-associated membranes [48].

Additional types of regulated cell death (e.g., parthanatos, immunogenic cell death, etc.) have recently been associated with myocardial ischemic injury and have been discussed in depth in specialized reviews [31].

## 3. The Role of Inflammation in Ischemic Injury

**Figure 2:**
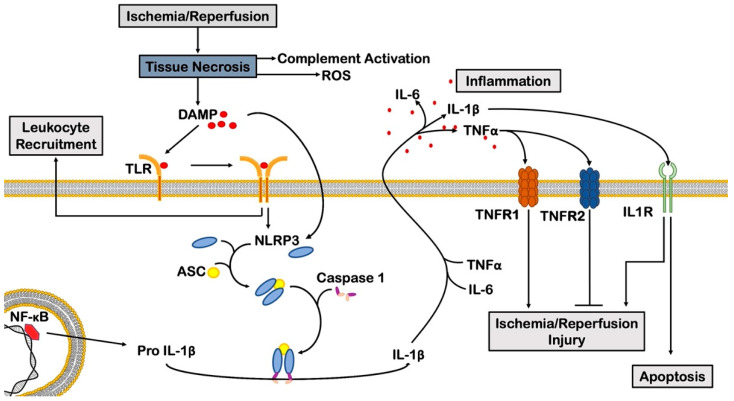
Schematic representation of the main signaling contributing to inflammation during myocardial ischemia. The inhibition of pro-inflammatory cytokines represents a useful therapeutic strategy to limit ischemic injury. ASC, apoptosis-associated speck-like protein containing a C-terminal caspase recruitment domain; DAMP, damage-associated molecular pattern; IL, interleukin; IL1R, interleukin 1 receptor; NF-κB, nuclear factor kappa-light-chain-enhancer of activated B cells; NLRP3, Nod-like receptor (NLR) family pyrin domain containing 3; ROS, reactive oxygen species; TNF(R), tumor necrosis factor (receptor); TLR, Toll-like receptor.

During acute myocardial ischemia, necrotic cell death triggers a pro-inflammatory response through several processes, including ROS production, complement cascade activation and damage-associated molecular patterns (DAMPs) release (Figure 2). DAMPs mediate cardiomyocyte death by binding Toll-like receptors (TLRs), recruiting leukocytes to the infarct zone and promoting the secretion of different pro-inflammatory cytokines [49]. Certain DAMPs, such as high mobility group box 1 (HMGB1), have a detrimental role in acute myocardial ischemia and a beneficial effect in the post-ischemic myocardium [50,51].

Downstream, the nucleotide-binding oligomerization domain-like receptor family pyrin domain containing 3 (NLRP3) inflammasome plays a central role in MI. The expression of the components of this multimeric complex (e.g., NLRP3, apoptosis speck-like protein (ASC) and caspase-1) was found to be enhanced in preclinical models of acute myocardial ischemia and chronic MI and in patients who died from acute myocardial ischemia [40]. In a model of I/R injury, KO mice deficient for ASC and caspase-1 showed decreased secretion of pro-inflammatory cytokines and reduced myocardial infarct size compared to wild-type mice [40]. During acute myocardial ischemia, NLRP3 is activated both in circulating inflammatory cells and in cardiomyocytes, contributing to interleukin (IL)-1β and caspase-1 release, respectively [52]. Inhibition of NLRP3 inflammasome was found to reduce infarct size and preserve cardiac function in various preclinical models of MI and I/R [53].

Fine coordination of muscle growth, inflammation, and angiogenesis are pivotal to ensure adaptive remodeling, whereas an alteration of this equilibrium causes the deterioration of cardiac structure and function. Several cytokines and growth factors play a major role among various molecular factors participating in left ventricular (LV) remodeling in response to hemodynamic overload and ischemic injuries. Among pro-inflammatory cytokines, TNFα, IL-1 and IL-6 are critical players in mediating myocardial ischemic injury.

### 3.1. TNF-Alpha

TNFα was found to be increased in the non-infarcted area after chronic MI. Increased survival, reduced cardiac rupture, decreased inflammatory cell infiltration and cytokine expression were observed in TNFα knockout mice (TNFα −/−) after chronic MI. Reduced cytokine expression was also associated with decreased activity of matrix metalloproteinase (MMP)-9 in the infarcted myocardium. In chronic MI, TNFα −/− mice show an improvement of cardiac function along with reduced apoptosis and fibrosis in the non-infarcted area [54]. In the same line of evidence, TNFα −/− mice undergoing myocardial I/R injury show reduced infarct size, decreased activation of nuclear factor-kappaB (NF-kB) and less leukocyte infiltration, due to a reduced expression of chemokines and adhesion molecules [55]. These results suggest that TNFα inhibition may represent a valid strategy to reduce I/R injury in addition to its beneficial effects in the reduction of cardiac hypertrophy and its maladaptive transition to heart failure. However, cytoprotective effects of TNFα during I/R were also reported. Mice carrying a double knockout of TNFα receptors (TNFR)-1/2 show a larger infarct size and increased apoptosis after acute myocardial ischemia [55]. Monden et al. further investigated the specific role of TNFα receptors R1 and R2 in chronic MI. Four weeks after ligation, they observed the same infarct size in R1 and R2 knockout animals compared to wild-type mice [56]. Interestingly, while R1 KO mice display improved contractile function and increased survival after infarction, R2 KO show exacerbated ventricular dysfunction and remodeling compared to R1 KO and wild type. Myocardial R1 expression levels are increased in R2 knockout animals in response to chronic MI. These results suggest that TNF-α exerts detrimental effects via R1 and exerts a protective role through R2 signaling [56]. In line with this evidence, TNFα was also associated with adaptive effects in overloaded hearts. In this condition, placental growth factor (PlGF) regulates vessel dimensions and promotes reparative cardiac inflammation by finely tuning the tissue inhibitor of metalloproteinases 3 (TIMP-3)/TNFα converting enzyme (TACE) axis. This ensures an adequate inflammatory milieu for adaptive cardiac hypertrophic remodeling [57].

### 3.2. IL-1

IL-1 isoforms alpha and beta are also involved in the inflammatory response to myocardial ischemia. Overexpression of the naturally occurring IL-1 receptor antagonist (IL-1RA) reduces myocardial damage and apoptosis after I/R, suggesting that IL-1α and IL-1β play detrimental effects during acute myocardial ischemia [58]. IL-1 receptor KO mice undergoing I/R show reduced fibrosis in the non-infarcted area along with decreased matrix metalloproteinase (MMP)-2 and MMP-3 expression in the peri-infarct area [59]. In contrast, the expression of IL-1β quickly increases after the acute phase of myocardial ischemia and its inhibition is detrimental, delaying scar formation and promoting cardiac rupture [60]. Macrophages are key mediators of inflammation during ischemic injury, contributing to the secretion of IL-1β. Javaheri and colleagues reported an impairment of lysosomal function in macrophages of mice subjected to cardiac IR injury. In the same study, the authors demonstrated that transcription factor EB (TFEB) overexpression in macrophages reduces myocardial IL-1β and improves cardiac function in mice undergoing I/R. TFEB is a transcriptional regulator of autophagy and lysosomal function. Mechanistically, the benefits induced by TFEB overexpression were found to rely on lysosomal lipolysis [61]. This study highlights important crosstalk between lysosomal function and inflammation.

### 3.3. IL-6

IL-6 was also reported to play both detrimental and protective effects during acute myocardial ischemia. Depending on the duration of ischemia, IL-6 KO mice were reported to show similar or smaller infarct size compared to wild-type mice [62,63,64], whereas IL-6 inhibition by the monoclonal antibody (MR16-1) administration before reperfusion worsened cardiac remodeling [65].

In conclusion, the inflammatory response is essential for adaptation to stress and survival after ischemic injury. However, excessive levels of inflammation, mainly due to aberrant cytokine upregulation, may contribute to ischemic injury and eventually lead to chronic maladaptive myocardial remodeling and heart failure. From a translational point of view, therapeutic approaches mitigating the levels of those cytokines that were proven to be detrimental in this pathological setting may represent the best strategy to grant survival in acute myocardial ischemia and preserve cardiac mechano-elastic properties in the long term (see Section 8.1 for details). However, an excessive reduction of inflammation should be avoided particularly in the acute phase of MI, since this may affect scar formation and may predispose to cardiac rupture.

Future studies should better clarify the involvement of inflammation in the setting of myocardial ischemia. In fact, surgical animal models of ischemia are often characterized by a confounding activation of inflammation due to the surgical introduction of sutures. To circumvent the inflammatory response associated with thoracotomy, closed-chest I/R models may be chosen [66].

## 4. The Role of Cell Metabolism in Myocardial Ischemia

Physiological mechanoelectrical activities of the heart require massive amounts of energy. Every day myocardial cardiomyocytes, which represent around 30% of total myocardial volume, consume an amount of ATP up to 20 times the whole heart mass.

Plasma levels of different substrates determine the specific substrates used by the heart for energy production, specific transporters on cardiomyocyte sarcolemma (e.g., monocarboxylate transporter (MCT) 1/2 for lactate and ketone bodies, Cluster of Differentiation (CD) 36 for fatty acids, glucose transporter (GLUT) 1–4 for glucose), metabolic enzyme availability (e.g., hexokinase, creatine kinase) and neurohormonal stimulation, with a prominent role for insulin. In this regard, increased insulin levels before ischemia were associated with increased MI [67,68].

Besides nutrient uptake alterations, myocardial ischemia leads to several metabolic derangements that depend on the extent and duration of the ischemic event. Cardiomyocyte damage may be reversible, causing temporary functional alterations (e.g., myocardial stunning and hibernation), or irreversible. Both reversible and irreversible myocardial damage were observed to correlate with mitochondrial dysfunction. A decline in state 3 oxygen consumption and respiratory control index in malate and succinate has been observed in several animal models of MI, together with a reduction of phosphocreatine and ATP levels [69]. Interestingly, interference with succinate metabolism by malonate administration protects the heart in murine and porcine models of I/R compared to controls [70,71]. Administration of beta-hydroxybutyrate, one of the main ketone bodies, before I/R or during reperfusion also exerts a protective effect by improving mitochondrial function [72,73].

### 4.1. Glycolytic Switch

In the presence of oxygen depletion, cardiomyocytes are forced to meet their energetic requirements by enhancing glycogenolysis and switching from fatty acid beta-oxidation to anaerobic glycolysis by adenosine monophosphate (AMP)-mediated protein kinase (AMPK) activation and by reduction of citrate-mediated 6-phosphofructokinase-1 (PFK-1) inhibitory function towards this process [74]. AMPK-deficient mice display more severe intracellular metabolic derangements and myocardial injury in response to I/R than wild-type controls [75]. In addition, autophagy may be suppressed in this condition since AMPK represents an important autophagy activator (see below). On the other hand, AMPK pharmacological reactivation reduces ischemic damage in the perfused heart [76]. These results suggest that multiple mechanisms are affected by AMPK deregulation. In addition, the translocation of hexokinase II (mt-HKII) to mitochondria was found to increase glycolysis and mitochondrial protection [77]. An impairment of this process was proposed as a possible explanation for the detrimental effects of high circulating fatty acids in models of I/R injury [78]. On the other hand, glucose appears to be an indispensable substrate for energy production in the ischemic heart. Previous seminal studies showed that as long as a low-flow (5%) is maintained in the infarcted myocardium, the observed damage is up to 20-fold less severe than a no-flow condition in ex vivo ischemia models. However, glucose deprivation in the perfusion mixture blunted the low-flow protective effects, demonstrating that cardiomyocytes can hardly benefit from other metabolic substrates in stress conditions [79,80].

The glycolytic switch causes lactate accumulation and cytoplasmic acidification, together with the accumulation of glycolytic by-products, which usually self-limit the entire process. However, during acute MI in patients, glycolytic by-products are washed away by residual blood flow, thereby keeping glycolysis highly active [81]. Inhibition of Na^+^/H^+^ exchanger isoform 1 (NHE1) by empagliflozin administration delays the onset of ischemic myocardial contracture in murine isolated hearts undergoing I/R [82]. In addition, empagliflozin inhibits sodium-glucose co-transporter 2 (SGLT2) and improves cardiac function after permanent coronary artery ligation in rats [83].

Several studies also focused on the potential efficacy of nutrient uptake modification to exert a protective effect in acute myocardial ischemia models. Glucose transporters (GLUTs) have been objects of intense study. In mice, chronic GLUT inhibition with ritonavir causes resistance to I/R due to metabolic adaptation in cardiomyocytes, which upregulates fatty acid transporter CD36 and fatty acid transcriptional regulator peroxisome proliferator-activated receptor α (PPARα) [84]. Conversely, ex vivo administration of a dopamine receptor D4 (DRD4) agonist through a Langerdorff system was found to promote GLUT4 translocation on the sarcoplasmic membrane in cardiomyocytes, exerting phosphatidylinositol 3-kinase (PI3K)/Protein kinase B (Akt)-dependent cardioprotective effect against simulated I/R [85]. Glucose consumption through glycolysis also affects the hexosamine biosynthesis pathway (HBP), which is altered by stress and nutrient availability. HBP is responsible for the O-linked attachment of N-acetylglucosamine moiety (O-GlcNAc), a post-translational modification of serine/threonine residues in proteins. An increase of this protein modifications by O-GlcNAc transferase (OGT) [86], or by inhibition of β-N-acetylglucosaminidase (OGA), was found to reduce myocardial injury in several models of I/R injury [87,88]. O-GlcNAcylation was also associated with better calcium handling [89], reduced mPTP opening and increased recruitment of the antiapoptotic protein Bcl-2 on the mitochondria, even though a further comprehension of these mechanisms is still required [90].

### 4.2. Fatty Acids and Ischemic Injury

Numerous studies on the role of fat as a metabolic substrate for energy production during myocardial ischemia have been conducted in recent years. Interestingly, hearts harvested from non-obese mice fed with a high-fat diet (HFD) show signs of altered calcium handling, mitochondrial morphology and oxidative state, and are more susceptible to in vitro I/R stress [91]. A similar result was obtained with obese rats, which display reduced mitochondrial biogenesis and ROS handling and have a worse cardiac function after I/R [92]. Diabetic and obese insulin-resistant patients have an increased risk of ischemic events and a worse injury [93,94]. Chronic subclinical inflammation also contributes to a worse cardiovascular outcome of these patients [95]. This is also supported by the evidence that type II diabetes, but not type I diabetes, abrogates cardioprotection provided by preconditioning [96]. Conversely, a recent study demonstrated that HFD increases myocardial ischemic tolerance through a delayed normalization of the pH, as long as mitochondrial redox uncoupling is kept under control in mice undergoing I/R [97]. In general, it seems that circulating fatty acid levels are critical determinants of whether these are protective or harmful [98].

**Figure 3:**
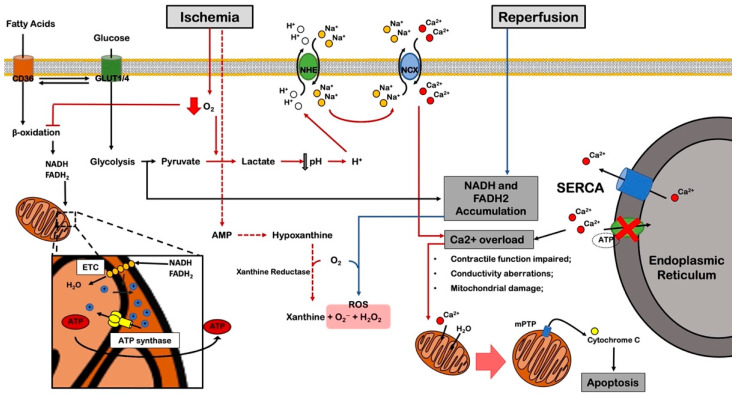
Schematic representation of molecular pathways regulating cell metabolism during myocardial ischemia. Ischemic injury leads to metabolic derangements, which converge to mitochondrial dysfunction and contractile dysfunction. AMP/ATP, adenosine mono/tri-phosphate; ETC, electron transport chain; FADH2, (reduced) flavin adenine dinucleotide; GLUT, glucose transporter; mPTP, mitochondrial permeability transition pore; NADH, (reduced) nicotinamide adenine dinucleotide; SERCA, sarco-endoplasmic reticulum calcium ATPase.

In conclusion, ischemic cardiomyocytes benefit from the upregulation of glycolysis (Figure 3) (e.g., through AMPK activators, insulin, metformin, adenosine, nicotinamide mononucleotide) for the adaptation to ischemia. However, a prolonged anaerobic energy supply appears to promote mitochondrial dysfunction and exacerbate I/R damage, as discussed in the following sections.

## 5. The Role of ROS in Myocardial Ischemia

**Figure 4:**
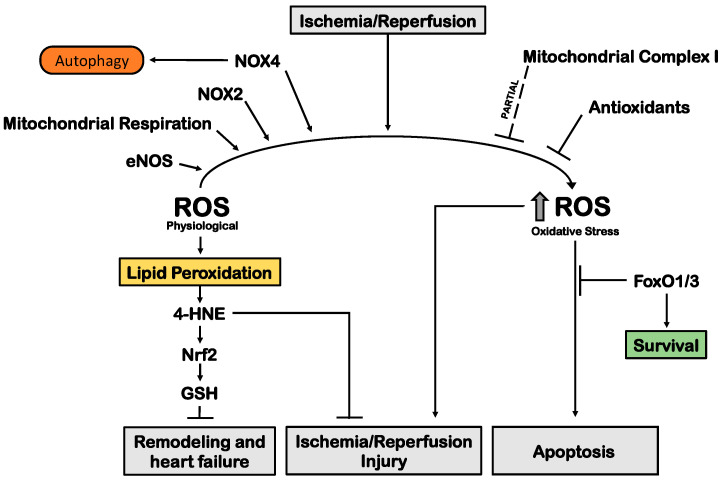
Schematic representation of the main pathways contributing to oxidative stress during myocardial ischemia. Physiological levels of ROS preserve cardiac function, whereas their increase enhances ischemia/reperfusion injury. 4-HNE, 4-hydroxynonenal; eNOS, endothelial nitric oxide (NO) synthase; GSH, glutathione; FoxO1/3, Forkhead Box O1; NOX2/4, nicotinamideadenine-dinucleotide phosphate (NADPH) oxidase 2/4; Nrf2, NF-E2–related factor 2; ROS, reactive oxygen species.

Reactive oxygen species (ROS) act as important signaling molecules in the cardiovascular system while within the physiological range levels. However, ROS overproduction represents one of the main pathophysiological events contributing to MI progression and I/R injury (Figure 4) by damaging lipids and membrane, proteins and nucleic acids [99,100]. Moreover, the oxygen supply that occurs during reperfusion after an ischemic episode exacerbates oxidative stress [101,102]. Antioxidant systems also appear to be impaired during myocardial ischemia, while antioxidant therapies improve cardiac function, both in animals and humans. Shiomi et al. reported that the overexpression of glutathione peroxidase (GSHPx) in mice prevents adverse remodeling and heart failure after chronic MI [103]. Similarly, administration of dimethylthiourea (DMTU), a hydroxyl radical scavenger, improves cardiac function in mice undergoing chronic MI [104].

ROS include the superoxide anion (O_2_^−^), the hydroxyl radical (OH) and hydrogen peroxide (H_2_O_2_) that are generated as by-products of different processes, such as mitochondrial respiration and endothelial nitric oxide synthase (eNOS) uncoupling [100].

### 5.1. Non-Reperfused Chronic Myocardial Infarction

Compelling evidence suggests that nicotinamide adenine dinucleotide phosphate hydrogen (NADPH) oxidase (Nox) isoforms play a fundamental role in ROS generation during chronic MI, worsening cardiac remodeling [105,106]. Systemic Nox2 knockout mice are protected from adverse cardiac remodeling and contractile dysfunction following chronic MI [107], whereas cardiac-specific Nox2 overexpression exacerbates post-infarction remodeling [108]. The link between Forkhead box O (FoxO) transcription factors and ROS during MI has also been highlighted. In vitro, FoxO1 and FoxO3 improve cardiomyocyte cell survival under oxidative injury. Similarly, combined cardiac-specific deletion of FoxO1 and FoxO3 decreases cardiac function at 4 weeks post-chronic MI [109].

### 5.2. Acute Myocardial Ischemia

Increased expression of Nox2 and Nox4 was reported in cardiac I/R models, along with marked ROS generation and mitochondrial dysfunction [110,111]. In fact, systemic and cardiac-specific Nox4 knockout mice show reduced I/R injury [110,112,113]. Crosstalk among Nox4, ROS and eNOS uncoupling during I/R injury has been described. I/R increases cardiac expression of Nox4 as a result of loss of nitric oxide (NO) bioavailability [111]. Rescue of NO bioavailability during I/R limits cardiac damage by decreasing Nox4 expression and eNOS uncoupling [111,114].

In contrast, combined systemic deletion of Nox2 with cardiac-restricted deletion of Nox4 exacerbates I/R injury, suggesting that physiological levels of ROS exert protective effects. Similarly, during acute myocardial ischemia without reperfusion, Nox4 expression and activity increase in the cardiac endoplasmic reticulum, thereby enhancing autophagy [78]. The latter suggests that Nox4 also triggers beneficial adaptive mechanisms, such as autophagy [99]. It has also been shown that Nox4 plays physiological effects in response to I/R injury. Santos and colleagues demonstrated that Nox4 is activated during I/R, exerting pro-survival mechanisms in cardiomyocytes through the activation of eIF2α /ATF4 signaling [115]. Overall, these data suggest that both excessive activation and suppression of Nox proteins, compared to physiological levels, may contribute to I/R injury, particularly if they alter adaptive signaling mechanisms [116].

Besides Nox, other mechanisms are involved in ROS production during I/R, such as those involving defective mitochondrial complex I (C-I) components. Interestingly, a study outlined that partial inhibition of mitochondrial C-I activity reduces I/R injury, whereas a complete inhibition exacerbates it [117]. Mitochondrial ROS (mtROS) are mainly produced by reverse electron transfer from complex (C)-II to C-I during early reperfusion. Inhibition of C-II reduces I/R injury in mice [118], in association with reduced ROS production and mPTP opening [71]. These results suggest that inhibition of reverse electron transport represents an effective strategy to reduce oxidative stress during I/R [71]. mtROS produced during I/R also promote apoptosis through the mitochondrial pathway and by the opening of mPTP in the inner mitochondrial membrane [119]. ROS also trigger lipid peroxidation, which results in the generation of α,β-unsaturated aldehydes, such as 4-hydroxy-2-nonenal (4-HNE). However, at low doses, 4-HNE confers cardioprotection. Zhang et al. demonstrated that 4-HNE treatment reduces I/R injury in isolated hearts through activation of nuclear factor-erythroid-2–related factor 2 (Nrf2), which in turn activates antioxidant pathways, e.g., glutathione (GSH) biosynthesis [120]. Combined cardiac-specific deletion of FoxO1 and FoxO3 increases I/R injury in mice. The detrimental effects of FoxO1 and FoxO3 in vivo are associated with increased cardiac oxidative stress and reduced expression of antioxidants [109].

In summary, current evidence highlights the harmful effect of increased ROS production deriving from the cellular alterations underlying MI and I/R injury. However, low physiological levels of ROS are required to preserve protein integrity and basal signaling. Although interventions aimed at reducing oxidative stress would be beneficial in ischemic injury, they should not affect physiological redox signaling and should always be coupled with interventions attenuating inflammation and metabolic alterations, as discussed below.

## 6. The Role of Autophagy in Myocardial Ischemia

Autophagy is one of the primary mechanisms ensuring cardiac homeostasis in response to stress, thus limiting cardiac damage and preserving heart function [121]. Autophagy is an evolutionary conserved intracellular catabolic pathway that removes damaged cytoplasmic cargoes such as senescent proteins or whole organelles [122,123,124]. Autophagy is generally activated by cellular stress, such as depletion of nutrients, mitochondrial damage, endoplasmic reticulum (ER) stress and oxidative stress. Among the main regulators of autophagy, mammalian target of rapamycin (mTOR) acts as an autophagy inhibitor, whereas the 5′-AMP-activated kinase (AMPK) and glycogen synthase kinase-3 beta (GSK-3β) are positive regulators [125,126,127].

In the heart, autophagy is involved in cardiac remodeling, limits ischemia/reperfusion (IR) injury and reduces cardiac complications induced by metabolic disorders, e.g., diabetes and obesity [121,128]. Generally, decreased autophagy is associated with cardiac aging and damage, whereas its stimulation confers beneficial effects. However, several studies have shown that excessive autophagy can trigger harmful mechanisms in the heart in some circumstances, increasing damage [121].

### 6.1. Acute and Chronic Ischemic Injury

**Figure 5:**
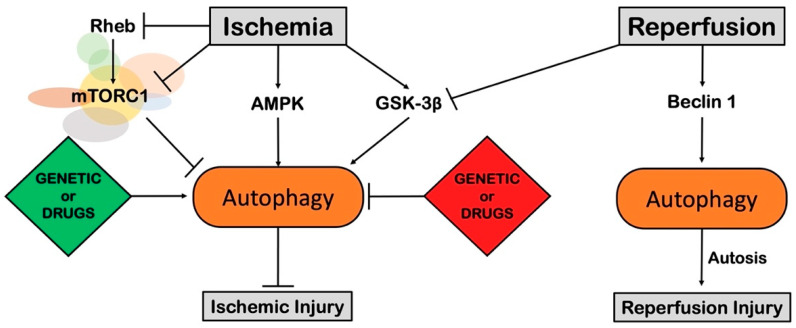
Schematic representation of the role of autophagy in myocardial ischemia. Autophagy activation before and immediately after ischemia limits ischemic injury (left). However, excessive activation of autophagy during reperfusion leads to detrimental effects. AMPK, adenosine monophosphate (AMP)-activated protein kinase; GSK-3β, glycogen synthase kinase 3β; MST1, mammalian sterile 20-like kinase 1; mTORC1, mammalian target of rapamycin complex 1; Rheb, Ras homolog enriched in brain.

The role of autophagy has been extensively studied in pre-clinical models of chronic and acute myocardial ischemia (Figure 5). Autophagy is activated during acute myocardial ischemia through AMPK activation and Ras homolog enriched in brain (Rheb)/mTOR complex 1 (mTORC1) inhibition. Several lines of evidence suggest that autophagy activation during myocardial ischemia is an adaptive response that limits cardiac injury. In fact, genetic inhibition of autophagy through AMPK inactivation or mTORC1 stimulation exacerbates cardiac injury in models of acute myocardial ischemia without reperfusion [129,130]. Conversely, boosting cardiac autophagy has been reported to limit injury and improve cardiac function. For example, starved mice showed reduced infarct size after acute myocardial ischemia [131]. In line with this evidence, autophagy stimulation by rapamycin, a mTORC1 inhibitor, was found to limit post-infarction cardiac remodeling and improve cardiac function in mice and rats that received permanent coronary artery ligation [132,133]. Different natural compounds able to stimulate autophagy were also demonstrated to reduce ischemic damage in models of myocardial ischemia [134]. The natural disaccharide trehalose reduces chronic infarction-induced cardiac remodeling and dysfunction in an autophagy-dependent manner. In fact, the protective effects of trehalose are attenuated in mice carrying a systemic heterozygous genetic deletion of Beclin 1 (Beclin1 +/−), a fundamental component of the autophagic machinery [135]. Similarly, the flavonoid 4,4′-dimethoxychalcone reduces the infarction area in wild-type mice subjected to prolonged myocardial ischemia and not in mice with cardiac-specific autophagy deficiency [136]. Other natural compounds able to stimulate autophagy, such as resveratrol and curcumin, were also found to improve cardiac function in chronic MI and I/R models, respectively [137,138]. However, the effects of resveratrol and curcumin in the presence of autophagy inhibition were not investigated, opening the possibility that other mechanisms may explain their protective effects in response to ischemic damage. The pro-apoptotic mammalian sterile 20–like kinase 1 (MST1) also plays an important role in regulating autophagy during chronic ischemic injury. This was first shown by Maejima and colleagues, who demonstrated that MST1 activation reduces cardiac autophagy and MST1 inhibition improves cardiac function and reduces infarct size in a model of chronic MI [139]. Overall, these results suggest that autophagy activation during acute and chronic myocardial ischemia plays a beneficial role.

### 6.2. Reperfusion Injury

The functions of autophagy in response to reperfusion injury are still debated. Autophagy is massively activated in the ischemic phase in an AMPK-independent manner, also exerting maladaptive effects (Figure 5). It was reported that Beclin 1 is activated at reperfusion and cardiac injury is reduced in Beclin 1 +/− mice [129]. Recently, it was suggested that the excessive activation of autophagy during the later phase of reperfusion triggers a peculiar form of cell death, named autosis, which is characterized by autophagy-dependence and non-apoptotic, non-necrotic features [140]. In a recent study, stimulation of autophagy by administration of the synthetic peptide Tat-Beclin 1 three hours after reperfusion exacerbated I/R injury, whereas inhibition of autosis attenuated myocardial damage. Mechanistically, I/R upregulates Run-domain Beclin 1 interacting and cysteine-rich-containing protein (Rubicon), leading to autophagosome accumulation and autosis. Genetic inhibition of Rubicon reduced I/R injury [141]. In line with these findings, Glycogen synthase kinase-3β (GSK-3β) in vivo inhibition was demonstrated to exacerbate myocardial damage in chronic ischemia and to protect from I/R injury by activating mTOR and consequently repressing autophagy [142]. However, it was recently shown that infusion of TAT-Beclin1 at the beginning of reperfusion improves heart function and reduces scar size after 24 h of I/R injury in wild-type mice. Tamoxifen-induced cardiomyocyte-specific autophagy-related (ATG)7 KO mice do not show improved recovery after TAT-Beclin1 infusion, demonstrating that the beneficial effects exerted by the peptide are autophagy-dependent [143]. Taken together, these results suggest that early autophagy activation after I/R exerts beneficial effects, but its late activation exacerbates I/R damage by Rubicon-mediated autosis activation.

### 6.3. The Specific Role of Mitophagy in I/R Injury

Mitophagy represents a fundamental pathophysiological mechanism during acute myocardial ischemia. The first clue was provided by observing that dysfunctional mitochondria are removed by increased mitophagy in the border zone of the infarct. Mice with genetic inhibition of mitophagy display larger infarct size than wild-type mice after permanent ligation of the left descending coronary artery [56]. In contrast to general autophagy, mitophagy exerts protective effects also during I/R. Dynamin-related protein 1 (DRP1) knockout mice show mitophagy inhibition, mitochondrial dysfunction and develop larger infarcts after I/R [144]. Mitophagy activation by simvastatin was found to reduce I/R injury in wild-type mice but had no effect in Parkin knockout mice, a model of genetic inhibition of mitophagy [145].

Overall, the evidence described above suggests that autophagy may be considered an attractive therapeutic target for managing ischemic injury. Further studies are still needed to better define future translational autophagy activation approaches in patients with MI to achieve cardioprotection without exerting maladaptive effects. Interventions aimed to stimulate autophagy may be considered protective during myocardial ischemia, as well as the use of small molecules able to target mitophagy selectively. Further strategies may be developed to specifically target autosis and prevent cardiomyocyte death, complementing those targeting necrosis and apoptosis during the late phase of reperfusion injury. To achieve this goal, new methods to better evaluate autophagy should be developed, especially in human samples.

## 7. The Role of Mitochondrial Dynamics in I/R Injury

Mitochondria are a cornerstone between ROS and cell metabolism in MI. Mitochondrial dynamics, i.e., the dynamic process of biogenesis, fusion, fission and degradation of these organelles, is increasingly emerging as a major player in cardiac homeostasis and pathology. Briefly, mitochondrial fission consists of the excision of a terminal part of the mitochondrion by a multimeric contractile ring composed of DRP1 GTPase proteins, recruited by specific localization proteins like mitochondrial fission protein 1 (Fis1), mitochondrial division protein 1 (Mdv1) and mitochondrial fission factor (MFF) [146]. Conversely, mitochondrial fusion consists of the confluence of both the outer and inner membrane of different mitochondria that are then fused into one. The process is complex and involves multiple proteins, including mitofusin (Mfn)-1/2, involved in the outer membrane fusion, and optic atrophy gene 1 (Opa1), involved in the inner membrane fusion [146].

The role of mitochondrial dynamics in ischemic injury has been broadly explored in vivo. Fission is usually activated after acute myocardial ischemia, and it was observed in several studies that its inhibition exerts protective effects. For instance, in a murine model of I/R, the pharmacological or adenoviral-mediated inhibition of DRP1 improves cardiac function and reduces infarct size [147,148]. In contrast, cardiac-specific DRP1 KO mice show greater infarct size in response to I/R because of impaired mitophagy flux. These results may suggest that physiological levels of fission should always be preserved [144].

By contrast, pharmacological induction of mitochondrial fusion reduced apoptosis and infarct size in a rat model of I/R [149], and Opa1 overexpression in mouse models of I/R was found to improve mitochondrial function [150]. In addition to its role in promoting mitochondrial fusion. Opa1 also participates in remodeling inner mitochondrial membrane (IMM) cristae. Its long form (L-Opa1) localizes to the IMM and undergoes proteolytic cleavage into short-Opa1 (S-Opa1). Proteolytic cleavage of L-Opa1 is induced by different mitochondrial metalloendopeptidases, among which the best characterized is Oma1. L-Opa1 cleavage by Oma1 promotes enhanced mitochondrial fragmentation, alteration of cristae morphology and induction apoptosis [151,152]. In isolated rat hearts undergoing I/R, increased proteolytic cleavage of L-Opa1 by Oma1 was observed, along with mPTP opening [153]. Mitochondrial swelling induced by Ca^2+^ was also reported to increase L-Opa1 cleavage in isolated rat heart mitochondria. Cells silenced for Opa1 are less sensitive to mPTP-induced swelling but are characterized by increased mitoROS and decreased ETC activity and oxidative phosphorylation [154]. These results suggest an important role of Opa1 in the regulation of mPTP opening. Mfn1/2 were also involved in I/R injury, even though additional studies are still required to clarify these molecules’ involvement in cardioprotection. Constitutive cardiomyocyte-specific Mfn2 KO mice display abnormal mitochondria and worse cardiac phenotype after I/R (30 min of ischemia + 30 min of reperfusion) [155]. Nevertheless, different studies found that constitutive cardiomyocyte-specific Mfn2-deficient mice have improved cardiac recovery after longer I/R (30 min of ischemia + 2 h of reperfusion) [156] and that Mfn2 in vitro overexpression is sufficient to induce apoptosis in cardiac cells [157]. This suggests that mitochondrial fusion is an adaptive response to early I/R injury, but its sustained activation may be detrimental in the long term. Mitochondrial biogenesis also represents a key mechanism during myocardial ischemia. Proliferator-Activated Receptor-Gamma Coactivator-1 alpha (PGC-1α) and β are the main transcriptional regulators of the heart’s mitochondrial biogenesis, metabolism and mitochondrial dynamics. In fact, systemic knockout models of PGC-1α and β develop cardiac dysfunction and heart failure and show mitochondrial abnormalities, such as fragmentation and elongation [158]. In the same line of evidence, in a model of chronic MI, the cardiac expression of PGC-1α, nuclear respiratory factor (NRF) -1, NRF-2, and melanotransferrin (MTF)-A decreases. Treatment with NHE-1-specific inhibitor rescues mitochondrial transcription factors expression and mitochondrial function. This suggests that targeting mitochondrial biogenesis may represent a strategy to improve cardiac function during myocardial ischemia [159].

**Figure 6:**
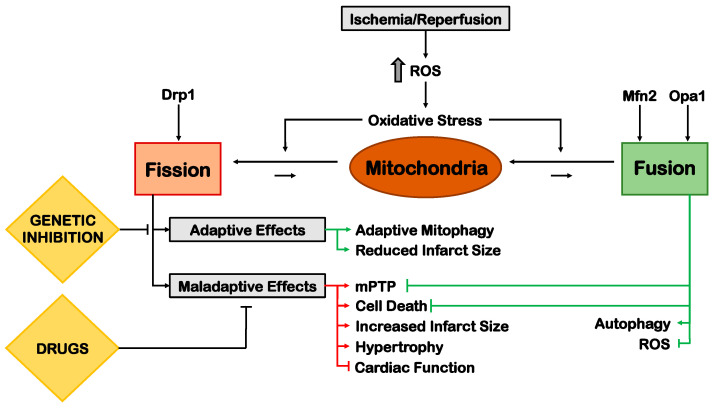
The role of mitochondrial dynamics in myocardial ischemia. Pharmacological inhibition of fission or fusion activation reduces cardiac injury. DRP1, dynamin-related protein 1; Mfn2, mitofusin 2; mPTP, mitochondrial permeability transition pore; Opa1, optic atrophy gene 1; ROS, reactive oxygen species.

In summary, mitochondrial dynamics should be preserved within physiological levels for appropriate cardioprotection. Ischemic injury shifts the process towards fission, which can be reduced or counteracted by stimulating fusion (Figure 6). Pharmacological intervention is already providing the first encouraging results in this novel field of research in myocardial ischemia and our understanding may be sufficient to verify the translation potential of these approaches with a clinical trial soon.

## 8. Clinical and Translational Perspectives

The above-described evidence suggests that alteration of different molecular mechanisms contributes to worsened cardiac function in response to ischemic injury. To date, several pharmacological compounds have been reported to reduce I/R injury. Drugs administered at the onset of myocardial reperfusion include antioxidants, pharmacologic antagonists of the sarcolemmal Ca^2+^ channel or the mitochondrial Ca^2+^ uniporter. While such strategies were reported to be effective in pre-clinical models, conflicting results were shown in clinical studies [160]. In addition, mPTP inhibitors, such as cyclosporine A, were found to reduce infarct size in pre-clinical models of I/R and in humans [160]. Exenatide, an analogue of glucagon-like peptide-1 (GLP-1), was reported to reduce myocardial infarct size and to improve myocardial salvage in patients with ST-segment elevation myocardial infarction (STEMI) [161].

The following section describes novel and perspective cardioprotective strategies for managing I/R injury and MI in detail.

### 8.1. Targeting of Inflammatory Mediators

Inhibiting inflammatory mediators represents a valid therapeutic approach to treating ischemic injury [162]. To date, different clinical trials have been performed; in particular, targeting IL-1 and IL-6 signaling. In two clinical trials, Abbate and colleagues showed that anakinra, an IL-1 receptor antagonist, improved LV remodeling in a small cohort of patients with acute STEMI. However, it failed to show a statistically significant effect on indexed left ventricular and systolic volume (LVESV), left ventricular and diastolic volume (LVEDV) or left ventricular ejection fraction (LVEF) in a larger cohort [163]. In a recent study in 99 patients with STEMI, anakinra reduced death, new-onset heart failure, death and hospitalization for heart failure [164]. Recently, in assessing the effect of Anti-IL-6 treatment in a myocardial infarction (ASSAIL-MI) trial, IL-6 inhibition by tocilizumab was reported to increase myocardial salvage in patients with acute STEMI [165].

### 8.2. Pre and Post-Conditioning

**Figure 7:**
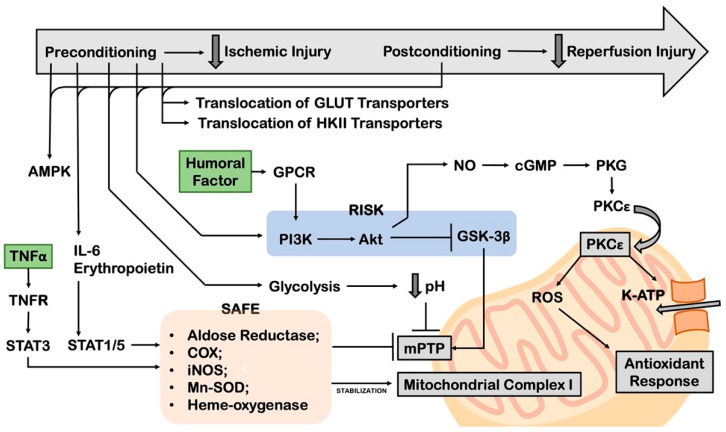
Schematic representation of the effects of ischemic preconditioning and post-conditioning during ischemia-reperfusion. The restoration of glycolysis and NO metabolism inhibit mPTP opening and reduce oxidative stress, improving mitochondrial function. AMPK, adenosine monophosphate (AMP)-activated protein kinase; COX, cyclooxygenase; cGMP, cyclic guanosine monophosphate (cGMP); GLUT, glucose transporter; GSK-3β, glycogen synthase kinase 3β; GPCR, G-protein coupled receptor; HKII, mitochondrial hexokinase II; IL, interleukin; iNOS, inducible nitric oxide synthase; mPTP, mitochondrial permeability transition pore; NO, nitric oxide; PI3K, phosphatidyl-inositol 3-kinase; PKC, protein kinase C; PKG, protein kinase G; RISK, reperfusion injury salvage kinase; SAFE, survivor activating factor enhancement; SOD, superoxide dismutase; STAT, transducer and activator of transcription; TNF(R), tumor necrosis factor (receptor).

To date, the most efficient approach to mild IRI is ischemic preconditioning (IPC), an intervention that was first developed in the 1980s by Murry CE et al. [166]. This term refers to applying cycles consisting of short periods of ischemia and reperfusion before inducing prolonged ischemic injury. IPC leads to infarcts up to 75% smaller in size than those observed in the control group. The molecular mechanism underlying this protective effect was found to be related to the pre-activation of aerobic glycolysis, the translocation of GLUT transporters to the membrane, the translocation of HKII to the mitochondria and the activation of AMPK and Akt [167] (Figure 7). The prominent role of glucose metabolism was highlighted in a study that demonstrated no protective effects for IPC in the absence of this monosaccharide [168]. In addition, IL-6 type cytokines and erythropoietin levels increase during IPC. They bind to glycoprotein (gp) 130 receptors, activate signal transducer and activator of transcription (STAT)-1 and -5 signaling pathways and upregulate aldose reductase, cyclooxygenase (COX), inducible nitric oxide synthase (iNOS), manganese-dependent superoxide dismutase (Mn-SOD) and heme oxygenase [169,170]. Similarly, TNFα receptors activate STAT-3 and promote the expression of the same stress-response proteins induced by IL-6 type cytokines, and they are together referred to as the ‘survivor activating factor enhancement (SAFE)’ pathway [171]. Besides, many different humoral factors (e.g., adenosine, bradykinin, catecholamines, opioids, prostaglandins [169]) bind to specific G protein-coupled receptors (GPCR) in response to IPC, which then converge on the NO/cGMP-dependent protein kinase (PKG) pathway [172] and on the so-called ‘Reperfusion Injury Salvage Kinase (RISK)’ pathway, which is mediated by the PI3K/Akt/GSK-3B axis [173]. Both the SAFE and RISK pathways operate on mitochondria. The SAFE pathway stimulates electron transport chain complex I and represses mPTP opening, but the exact underlying mechanism still needs to be defined [174,175]. On the other hand, the RISK pathway inhibits GSK-3B signaling, thereby inhibiting mPTP opening [176]. Moreover, the NO/PKG pathway activates PKCε, which translocates to mitochondria, where it constitutes a positive feedback signalosome with PKG, ATP-dependent potassium channel (K-ATP) and ROS, stimulating an antioxidant response [177]. In addition, pioneering studies showed that mPTP mediates the effects of IPC. For example, IPC was reported to inhibit mPTP opening in reperfused rat hearts [178]. IPC induces transient mPTP (low-conductance) opening through different mechanisms. These include mitochondrial uncoupling, ROS signaling and augmentation of the pH of the mitochondrial matrix, which together protect the heart by reducing mitochondrial calcium load and facilitating ROS signaling [179].

IPC is not usually a feasible approach for most cardiac derangements in clinical practice. To overcome this issue, it was demonstrated that three cycles of 30 s reperfusion/30 s re-occlusion before complete reperfusion exert the same beneficial effects of IPC. This intervention was named ‘ischemic post-conditioning’ (IPoC) [180] and was found to reduce reperfusion injury in patients with STEMI undergoing percutaneous revascularization [181]. Post-conditioning was found to be associated with the same signaling and metabolic signature observed in IPC, including increased glycolysis and the maintenance of a low pH, which in turn prevents mitochondrial permeability transition pore (mPTP) opening [182].

Of note, remote ischemic conditioning (RIC) also recapitulates the beneficial effects of IRC and can be performed before, during or after the infarction onset. It consists of short cycles of ischemia-reperfusion in a remote area that can reduce myocardial infarct size after a prolonged coronary occlusion. In the first description of this phenomenon, Przyklenk et al. elegantly showed that short cycles of occlusion and reperfusion of the left circumflex coronary artery could reduce anterior infarct size induced by left anterior descending artery (LAD) occlusion [183]. Subsequent experimental works found that short ischemia and reperfusions cycles in peripheral skeletal muscles could attenuate subsequent myocardial I/R injury. This approach was demonstrated to be feasible in the clinical setting, in which short cycles of forearm ischemia and reperfusion through inflation and deflation of a sphygmomanometer cuff were found to reduce ischemic injury in patients with MI [184,185,186]. On the other hand, other large multicenter clinical trials did not demonstrate similar benefits in patients undergoing cardiac surgery [187], suggesting that the protective effects of RIC are context-dependent and warrant additional demonstration.

To date, a comprehensive explanation of how RIC exactly works has not been provided, but some molecular aspects have been clarified. For instance, the activation of sensory fibers in the peripheral organs triggers spinal and supraspinal reflexes, stimulating the autonomic innervation of the heart [188,189,190]. Moreover, the transfusion of ‘conditioned plasma’ from an animal that received ischemic conditioning to another animal was found to be able to protect the receiving animal during myocardial I/R injury, indicating that RIC elicits cardioprotection also through humoral mediators [191]. Many candidates have been identified (e.g., stromal cell-derived factor-1α [192], IL-10 [193], NO [194], miR-144 [195]) but none of them were sufficient to recapitulate RIC when administered alone. Considering that the crucial mediator of RIC beneficial effects was advocated to be hydrophobic and <15 kDa [169], we believe that the role of exosome cargoes should be studied thoroughly in the future.

### 8.3. Stem Cells and Exosome-Based Therapies

Stem cells have represented a fascinating approach to treating MI for a long time. However, multiple issues have limited their use in clinical practice so far. Among the potential issues affecting stem cell efficacy for cardiac repair, potential neoplastic transformation, undesired transdifferentiation, insufficient quantity, low survival after delivery, possible rejection in case of allografts and ethical concerns regarding embryo use, are the most relevant.

To date, skeletal myoblasts (SMs), mesenchymal stem cells (MSCs) and inducible pluripotent stem cells (iPSCs)-derived cardiomyocytes (iPSC-CMs) are those that best meet the technical requirements for myocardial regeneration. SMs were the first tested cell type, displaying good engrafting and reparative potential [196].

The phase I MAGIC clinical trial used autologous injections of SMs to treat patients with acute MI, but the procedure was considered unsafe due to the development of arrhythmia [197]. A possible improvement for the technology related to these readily available cells may be represented by transdifferentiating autologous SMs to CMs before implantation [198].

A large corpus of studies has been based on the intracoronary perfusion of autologous human bone marrow-derived mononuclear cells (hBMNCs) during percutaneous coronary interventions (PCI), as summarized by Higuchi et al. [199]. In brief, despite scar size being transversally reduced among the studies, most of these showed only a faint, or absent, improvement of LVEF and pathological remodeling after autologous hHBMNCs transfusion. A similar result was obtained with mononuclear peripheric blood cells (HEBE) [200] and CD34+ CXCR4+ (REGENT) [201]. In contrast, the use of bone marrow-derived mesenchymal stem cells (hMSCs) was safe and granted reverse remodeling, improved LVEF and reduced premature ventricle contraction (PVC) (PROCYMAL) [202]. Encouraging results were also achieved using different sources of MSCs, e.g., fetal human Wharton’s jelly (hWJ-MSCs) [203].

Cardiac interstitial cells also have regenerative potential. They include cardiosphere-derived cells (CDCs) and C-kit+ cells. The use of these cells was found to reduce ischemic injury and promote cardiac repair through multiple mechanisms, which have been reviewed elsewhere [204,205]. A combination of these cells with MSCs was shown to enhance beneficial cardiac effects and this approach was also tested in clinical trials [206,207]. Of note, a recent study proposed a novel, promising 3D structure composed of C-kit+ cells, MSCs and endothelial cells, that was called ‘CardioCluster’, as a potential candidate for cell therapies in MI [208].

Increasing lines of evidence also suggest that a paracrine effect largely mediates the beneficial effects of these approaches in most myocardial cell therapies, exceeding the impact of direct transdifferentiation of stem cells into healthy myocardium [209]. Encouraging results were obtained using stromal cells, such as adipose-derived stromal cells (ADSCs), whose beneficial effects were shown to be mediated by diffusible factors [210]. Consequently, much interest has grown around ADSCs-derived exosomes, which were proven to ameliorate mouse recovery after ischemia by M2 macrophage polarization [211]. The increase of local recruitment of ADSCs to the ischemic tissue by N-cadherin overexpression als0 improves the phenotype of infarcted adult mice in a beta-Catenin-dependent manner [212].

ADSCs are not the only source of exosomes studied in past years. Promising results were also observed in pre-clinical murine models of ischemic damage testing exosomes derived from endothelial progenitors [213], the exosome-rich plasma fraction [214], M2 macrophages [215], bone marrow-derived MSCs [216], CDCs [217], cortical bone stem cells [218], embryonic stem cells [219] and human inducible pluripotent stem cells (hiPSC) [220]. These studies mechanistically linked exosome-mediated protective effects with reduction of typical maladaptive processes in MI, such as excessive inflammation (counteracted by exosomes through integrin-linked kinase (ILK) inhibition [213], PKC*δ* downregulation, TLR-4 activation [215,217] and thioredoxin interacting protein (TXNIP) [215]) and autophagy impairment (recovered by exosomes through the mir125b-p53-Bnip3 pathway). It was observed that the anti-inflammatory IL-10 is downregulated in MI, with a detrimental effect on the secretory phenotype of the progenitor cells administered to the patients. However, targeting ILK was proposed as a novel approach to reduce the inflammation-mediated impairment of exosome-based therapies [213].

Several regenerative approaches based on human embryonic stem cells (hESCs) and human pluripotent stem cells (hPSCs) have been approved for clinical trials in recent years. Among these, the transplantation of bioengineered patches to treat acute MI provides encouraging results. In the ESCORT trial, a fibrin patch seeded with hESCs-derived cardiac progenitors was transplanted inside a subepicardial surgical pouch on patients with ischemic severe heart failure during coronary bypass. These interventions led to functional myocardial recovery and developed neither arrhythmias nor tumors. Alloimmunization was observed but remained clinically silent during the follow-up [221].

In the future, a challenge will be the improvement of techniques to better deliver stem cells or stem cell-derived paracrine factors to the damaged myocardium to promote functional recovery [222].

### 8.4. Bioengineering and Heart Reparation

Stem cell suspensions often aggregate and die, determining dramatic issues of cell survival, accurate delivery, durable engrafting and overall outcome. To overcome these difficulties, bioscaffolds have been developed to be seeded with stem cells and improve their delivery. However, several issues also affect the use of these products, such as duration of patch implantation on the myocardium, time of preparation, neo-vascularization of the scaffold, foreign body reaction, allogenic graft rejection, inflammation and arrhythmogenic potential of the patch.

#### 8.4.1. Cell Sheets

Many cell types have been tested to find a balance between the capacity to sustain neovascularization, provide structural robustness, form functional gap junctions, be immunologically tolerated, and availability for translational approaches.

In this sense, iPSCs are considered a promising candidate for the development of cell sheets since they are derived from easily accessible autologous cells, are highly plastic and proliferative, and their use is not limited to ethical issues (as for embryonic stem cells) [223,224].

The grafting of iPSC-CM sheets on murine and clinically relevant porcine models of MI displayed stable improvement of cardiac function, reduced fibrosis, neovascularization and cardiomyogenesis after 8–12 weeks, particularly when combined with the transplantation of an omentum delivering proangiogenic factors [225,226,227,228]. To date, this approach is considered the most promising and is being perfected by many groups around the world. Patches were also created employing bone-marrow-derived MSCs [229], menstrual blood [230] and ADSCs [231,232,233,234]. In all these MSCs-based models, cardiac function and tissue repair were improved after MI. The MAGNUM clinical trial explored the feasibility and safety of MI treatment using bone marrow-derived mononuclear cells (BM-MNCs) seeded onto collagen scaffolds grafted onto the infarcted ventricle of patients undergoing surgery. The results showed a general improvement in the efficiency of cellular cardiomyoplasty with increased viable tissue within the infarct scar. This resulted in positive remodeling with improved diastolic function [235].

Nevertheless, neither MSCs nor SMs displayed physiological electromechanical coupling with the host myocardium [236,237]. However, it was shown that patches realized with these cells still exert their protective effects by paracrine conditioning of the infarcted area [196,238], which is considered, to date, to be the main effector of cell sheet-based approaches in MI [239]. The secreted factors are mainly cytokines, exosomes and growth factors promoting neovascularization of the injured tissue and the implanted construct [240]. Although the exact nature of the involved secretome is still being defined, activation of the pro-angiogenetic PI3-kinase/Akt pathway was observed in several models [240]. Neoangiogenesis may also be stimulated by preparing patches with different cell types derived by iPSCs [241], including cardiomyocytes, endothelial cells and mural cells, a practice that resulted in better long-term performance of the construct in terms of systolic function, fibrosis, infarct wall thinning and neoangiogenesis in vivo (the latter being strongly dependent on the presence of CMs) [242]. A similar result was reached using a patch loaded with iPSC-CMs and MSCs in a rat model of chronic MI [243]. Interestingly, patches may be primed towards a neoangiogenic secretory phenotype before implantation, as demonstrated with a CDC-based sheet exposed to hypoxia (2% O_2_) for 24 h before grafting [244]. Administration of irbesartan, an angiotensin II receptor blocker, abrogates the protective role of an ADSC-derived cell sheet in a rat model of MI [245], suggesting a role of angiotensin-II in these processes and demonstrating the central role of diffusible factors in these treatments, opening the possibility of recapitulating the beneficial effects of patches by a direct administration of the relevant paracrine factors in vivo.

#### 8.4.2. Hydrogel Scaffolds

Scaffolds can be either pre-formed with a specific shape or prepared to take advantage of a state transition from liquid to solid, taking advantage of physical properties of the environment (e.g., pH, temperature) or biochemical reactions (e.g., polymerization). These types of scaffolds are usually referred to as ‘hydrogels’ and have been shown to significantly increase the therapeutic potential of cell therapies [246].

An unconventional approach was pursued by Huang et al., who recently developed a long-term storable acellular scaffold that improved myocardial recovery and function in a rat model of acute MI. In detail, the so-called artificial cardiac patch (artCP) was produced by embedding biodegradable polylactic-co-glycolic acid (PLGA) microparticles encapsulated with human cardiac stromal cell (CSC)-secreted factors, referred to as ‘synthetic CSCs’, into decellularized porcine myocardial scaffolds, promoting neovascularization and constructive remodeling [247].

It was also observed that viscoelastic patches exert their protective function in MI only for their mechanical properties, without any cell or factor embedded [248]. This suggests that approaches combining the extensive knowledge we have in cell sheet transplantation and scaffold bioengineering may produce substantial synergistic effects. From similar considerations, the novel field of 3D bioprinting has intersected with myocardial bioengineering and moved its first steps into an unexplored discipline of study. A 3D-printed scaffold using cardiac-derived progenitor cells embedded in a hyaluronic acid/gelatin (HA/gel)-based matrix recovered the phenotype of a mouse model of chronic MI [249]. Encouraging results were also obtained by use of Human Umbilical Vein Endothelial Cells (HUVECs) and iPSC-CMs encapsulated within hydrogel strands containing alginate and PEG-Fibrinogen (PF), which were deposited through a custom high-resolution microfluidic printing head (MPH) [250]. Besides patching, 3D printing has been used to reconstruct personalized sternal prostheses after invasive cardiosurgical procedures, construct personalized coronary models, create customized models of the patient’s anatomy, accurately plan surgical interventions and produce fully bioresorbable sulfated chitosan-modified stents, with promising results [251].

### 8.5. Mitochondrial Transplantation

In past years, the transplantation of viable and redox-competent mitochondria has been proposed to improve myocardial recovery after ischemic damage. The first evidence was provided by a rabbit model of 30 min ischemia followed by freshly isolated left-ventricular mitochondria injection from healthy donor’s hearts before 2 h of reperfusion [252]. A similar result was obtained with autologous transplantation of mitochondria that were derived from skeletal muscle and liver, from which a high yield of functional mitochondria, without immunologic issues, was obtained [253]. Treated animals displayed signs of cardioprotection, including a reduction in infarct size and inflammatory markers, increased ATP content, improved cardiomyocyte survival and cardiac function after MI. The mechanism of internalization of mitochondria is still under investigation, but endocytosis is considered a primary mechanism [254]. Interestingly, the mitochondria taken up might be intercellularly transferred along the myocardium through tunneling nanotubes [255]. However, the human translation of mitochondrial transplantation failed to provide consistent benefits, and further studies are still required to overcome the limits of this approach [256].

### 8.6. Robotics and Nanotechnology

Robotic surgery has been introduced in the field of cardiology, mainly for PCI [257], coronary artery bypass grafting [258], atrial fibrillation ablation and angiography [259]. The use of robotics reduces the amount of radiological exposure for the interventional cardiologist and grants increased ergonomics, visibility and precision under challenging settings. However, high costs and a steep learning curve limit the wide adoption of robotics for routine procedures in clinical practice. Currently, robotic approaches in cardiologic patients are mainly reserved for invasive procedures, e.g., valve repair/replacement and septal defect closures [260]. In the future, reducing size, economic accessibility and easiness of use will be required to spread hybrid [261] and fully-robotics approaches to routine activities.

Nanotechnology has also been shown to be potentially helpful for treating cardiovascular diseases. The term refers to the engineering of materials at the molecular level to develop novel desired properties. Liposomes and micellar nanoparticles have been used for drug delivery to improve target binding, reduce drug dosage and body clearance, and display biomimetic effects. For example, endothelial alpha(v)beta3 integrin-targeted fumagillin nanoparticles inhibit angiogenesis in atherosclerosis and stimulate plaque regression using a lower (one-third) dose of the drug, as compared to that borne by non-targeted nanoparticles showing a similar regressive effect on the plaque [262]. In the setting of MI, nanotechnology has been used mainly for stent coating in PCI. For instance, to achieve better endothelial healing after PCI, the first studies used nano-sized hydroxyapatite-coated with sirolimus [263] or magnetic silica nanoparticles loaded with rapamycin [264], restoring the vessels to a healthy condition. Similarly, nano-constructs carrying polycaprolactone [265] or alendronate [266] were found to avoid restenosis after stent implantation. From these seminal studies, novel ideas were developed around stent implantations, including the so-called ‘gene-eluting’ stents to carry antisense DNA and RNA oligonucleotides, DNA plasmids, peptides and also enzymes (e.g., nitric oxide synthase) [267]. Of note, a recent improvement for the treatment of I/R injury was achieved by using H_2_O_2_-responsive nanoparticles that are activated at the site of ROS overproduction, thus delivering potent on-target antioxidant [268] or anti-inflammatory activities [269]. An interesting application of nanomaterials to MI was to improve the arrhythmogenicity of collagen bio-scaffold patches by embedding them with conductive carbon nanofibers, thereby enhancing regeneration, neo-vascularization, immature cardiomyocyte proliferation and reducing fibrosis [270]. Improved performance of myocardial patches in MI was also obtained by boosting neoangiogenesis using a combined construct of a six-layered cardiomyocyte sheet and a poly(vinyl alcohol) fiber mat bearing poly(lactic-co-glycolic acid) nanoparticles that locally release vascular endothelial growth factor (VEGF) in an athymic rat model [271].

It is worth mentioning that nanorobotics is rapidly evolving from a theoretical field to an actual emerging R&D field, with the development of programmed nanomachines ranging from 0.1 to 10 μm. The application of these fascinating tools in cardiology may include drug delivery, mechanical blood clot enzymatic digestion and mechanic disruption [272].

It is hard to predict how nanotechnology will impact the future of clinical practice. An increasing number of integrated approaches will likely be developed along with the discovery of novel nano-constructs.

## 9. Conclusions

Currently, MI represents a significant concern for public health. Much effort is being devoted to reducing the impact of this pathology in terms of cost for national health systems and lost disability-adjusted life years (DALYs). The scientific community has been studying the molecular mechanisms underlying this severe disease for decades, and novel therapeutic approaches have been developed in recent years. However, further efforts should be made to fully elucidate the contribution of the major cell death mechanisms during ischemia and reperfusion. The exact time windows during which one specific form of cell death predominantly affects cardiac function and causes more severe injury is unclear. This would significantly facilitate intervention with therapies targeting specific molecular pathways. However, we believe that one of the strategies we mentioned, or a combination of more of these treatments, will prove to reduce the impact of MI on public health within the next decades.

## Data Availability

Not applicable.
